# Individualized Lung‐Protective Ventilation Strategy Based on Esophageal Pressure Monitoring in Patients With ARDS Associated With Severe Acute Pancreatitis—A Randomized Controlled Trial

**DOI:** 10.1002/wjs.12676

**Published:** 2025-07-25

**Authors:** Yang Zhao, Shengxuan Zhang, Chengjiang Liu, Xiaoxia Wang

**Affiliations:** ^1^ Shandong University of Traditional Chinese Medicine Jinan China; ^2^ Zhejiang Chinese Medical University Hangzhou China; ^3^ Department of General Medicine Affiliated Anqing First People's Hospital of Anhui Medical University Anqing China; ^4^ Department of Anesthesiology The Maternal and Child Health Hospital of Guangxi Zhuang Autonomous Region Guangxi China

**Keywords:** acute respiratory distress syndrome, esophageal pressure, individualized treatment, mechanical ventilation, severe acute pancreatitis, transpulmonary pressure

## Abstract

**Background and Objective:**

Acute respiratory distress syndrome (ARDS) secondary to severe acute pancreatitis (SAP) presents significant management challenges with high mortality rates. This study aimed to investigate the application value of an individualized lung‐protective ventilation strategy guided by esophageal pressure (Pes) monitoring in patients with ARDS associated with SAP.

**Methods:**

This randomized controlled trial included 124 patients with SAP‐related ARDS admitted to our hospital from January 2023 to December 2023, and they were randomized to a conventional lung protective ventilation group (conventional group, *n* = 62) and an esophageal pressure monitoring‐guided group (EPM‐guided group, *n* = 62). The conventional group adopted a conventional lung protective ventilation strategy; whereas, the EPM‐guided group received the individualized ventilation strategy based on EPM. The EPM indicators, respiratory mechanics parameters, oxygenation indicators, and clinical outcomes were compared between the two groups.

**Results:**

After treatment, the EPM‐guided group showed significantly lower transpulmonary pressure (PL) [(16.82 ± 2.46) versus. (22.41 ± 3.23) cmH2O, *p* = 0.006], transpulmonary driving pressure (ΔPL) [(12.36 ± 1.83) versus. (16.52 ± 2.37) cmH2O, *p* = 0.007], and driving pressure (ΔP) [(11.43 ± 1.83) versus. (14.52 ± 2.24) cmH2O, *p* = 0.008] than the conventional group, whereas static compliance (Cst) [(37.82 ± 4.46) versus. (29.41 ± 5.23) mL/cmH2O, *p* = 0.009] and the PaO2/FiO2 ratio [(268.82 ± 32.46) versus. (195.41 ± 28.23) mmHg, *p* = 0.008] were significantly higher. The EPM‐guided group had shorter mechanical ventilation duration [(12.32 ± 3.24) versus. (16.83 ± 4.52) d, *p* = 0.013] and intensive care nit (ICU) length of stay [(18.53 ± 4.62) versus. (23.72 ± 5.83) d, *p* = 0.018] compared to the conventional group, along with a lower VAP incidence (14.52% vs. 25.81% and *p* = 0.038) and a 28‐day mortality rate (19.35% vs. 32.26% and *p* = 0.042). Multivariate logistic regression analysis showed that ΔPL at 72 h (OR 1.56, 95% CI 1.25–2.01, *p* < 0.001) was an independent predictor of a 28‐day mortality rate. ROC curve analysis showed that ΔPL had a good diagnostic value for predicting a 28‐day mortality rate (AUC = 0.832 and 95% CI 0.760–0.904). Correlation analysis showed that ΔPL at 72 h was significantly negatively correlated with the PaO2/FiO2 ratio (*r* = ‐0.71 and *p* < 0.001) and static compliance (*r* = −0.69 and *p* < 0.001).

**Conclusion:**

Individualized lung protective ventilation strategy guided by EPM can more accurately assess the actual lung inflation pressure, optimize the setting of ventilation parameters, and improve clinical outcomes of patients with SAP‐related ARDS.

## Graphical abstract

Acute respiratory distress syndrome (ARDS) secondary to severe acute pancreatitis (SAP) presents significant management challenges with high mortality rates. This study aimed to investigate the application value of an individualized lung‐protective ventilation strategy guided by esophageal pressure (Pes) monitoring in patients with ARDS associated with SAP. This randomized controlled trial included 124 patients with SAP‐related ARDS admitted to our hospital from January 2023 to December 2023, and they were randomized to a conventional lung protective ventilation group (conventional group, *n* = 62) and an esophageal pressure monitoring‐guided group (EPM‐guided group, *n* = 62).

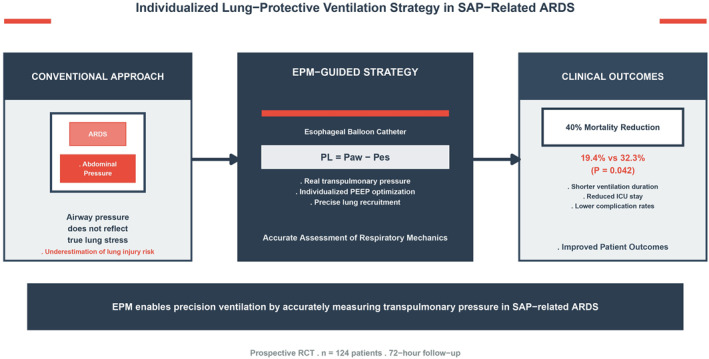

## 1 Introduction

Acute respiratory distress syndrome (ARDS) associated with severe acute pancreatitis (SAP) represents one of the most challenging clinical scenarios in critical care medicine. In patients with SAP, ARDS significantly increases mortality and prolongs hospitalization, representing a substantial clinical and economic burden. According to a cohort study by Zhang et al., ARDS and renal insufficiency are important predictors of early death in SAP patients and the overall mortality rate of SAP patients in their study was as high as 11.96% [1]. This highlights the urgent need for improved management strategies for SAP‐related ARDS.

The pathophysiological mechanisms of SAP‐induced ARDS involve systemic inflammatory responses, alveolar‐capillary barrier damage, and increased permeability leading to pulmonary edema. Importantly, patients with SAP frequently develop intra‐abdominal hypertension, which directly affects respiratory mechanics by elevating intrathoracic pressures [2, 3]. This creates unique challenges for mechanical ventilation management compared to ARDS from other etiologies.

Mechanical ventilation remains the cornerstone of supportive treatment for SAP‐ARDS, and the appropriate selection of ventilation parameters directly impacts patient outcomes [3]. In recent years, lung protective ventilation strategies have become standard practice for ARDS management. However, conventional lung protective ventilation strategies primarily rely on airway pressure monitoring to guide ventilation parameter adjustment, which has significant limitations [4]. Studies have shown that airway pressure may not accurately reflect the actual alveolar pressure, especially in patients with altered chest and abdominal mechanics [5].

This limitation is particularly relevant in patients with SAP. Due to intra‐abdominal hypertension, intrathoracic pressure increases significantly, meaning that ventilation strategies based solely on airway pressure may substantially underestimate the actual alveolar pressure [5]. Studies have demonstrated that under identical airway pressures, the actual transpulmonary pressure (PL) can vary considerably between patients, with these differences being more pronounced in patients with SAP [6]. This physiological reality underscores the need for individualized ventilation approaches that account for each patient's unique respiratory mechanics.

Esophageal pressure (Pes) monitoring has emerged as a valuable tool to address these limitations.

By measuring intrathoracic pressure in real time and dynamically, this technology can more accurately assess PL and transpulmonary driving pressure (ΔPL), providing a reliable basis for individualized ventilation parameter adjustment [7]. Recent studies have shown that ventilation strategies based on EPM can significantly improve oxygenation function and clinical outcomes of patients with ARDS [8].

Considering the specific pathophysiological characteristics of SAP patients with ARDS, including intra‐abdominal hypertension, pleural effusion, and pulmonary edema, we hypothesized that an individualized ventilation strategy guided by EPM would have particular clinical benefits in this population [9]. This approach may not only overcome the measurement limitations of conventional ventilation strategies but also allow precise parameter adjustment based on individual patient characteristics, potentially minimizing ventilator‐induced lung injury.

This study aims to evaluate the efficacy of an individualized lung protective ventilation strategy guided by EPM in patients with SAP‐related ARDS. We hypothesized that compared to conventional lung‐protective ventilation, this individualized approach would provide more accurate assessment of alveolar pressures, optimize ventilation parameters, and ultimately improve respiratory mechanics, oxygenation, and clinical outcomes in this high‐risk patient population.

## 2 Materials and Methods

### 2.1 General Information

This randomized controlled trial was conducted in the intensive care unit of Affiliated Anqing First People's Hospital of Anhui Medical University from January 1, 2023 to December 31, 2023. The hospital is a 1420‐bed tertiary care center with a 25‐bed mixed medical‐surgical intensive care unit (ICU). During the study period, our ICU admitted 876 patients requiring mechanical ventilation, of which 193 (22.0%) were diagnosed with acute pancreatitis and 138 (15.8%) with SAP according to the 2012 revised Atlanta criteria. The most common etiologies of SAP in our population were gallstone‐related (42%), alcohol‐induced (27%), hypertriglyceridemia (18%), and other causes (13%). The study protocol was approved by the Ethics Committee of Affiliated Anqing First People's Hospital of Anhui Medical University (AFky2023010322).

Sample size calculation was performed using the G*Power 3.1 software. Based on previous studies, we assumed a mean difference in the PaO2/FiO2 ratio of 50 mmHg between groups with a standard deviation of 80 mmHg at 72 h after intervention. With *α* = 0.05 and power = 0.90, the minimum required sample size was 54 patients per group. Considering a potential dropout rate of 15%, we planned to enroll 62 patients in each group. Although mortality would be an important outcome measure, we chose the PaO2/FiO2 ratio as our primary endpoint for sample size calculation due to its established use in previous similar studies and its direct relationship to the physiological effects of our intervention.

Eligible patients were screened within 48 h of mechanical ventilation and randomly assigned in a 1:1 ratio to either the EPM‐guided group or the conventional group using computer‐generated randomization. An independent research coordinator, uninvolved in clinical care or outcome assessment, performed the allocation using sequentially numbered, opaque, sealed envelopes to maintain concealment.

Inclusion criteria were as follows: (1) age ≥ 18 years, (2) meeting the diagnostic criteria for ARDS according to the 2012 Berlin Definition [10], (3) the cause of ARDS was SAP, meeting the 2012 revised Atlanta criteria [11], (4) requiring invasive mechanical ventilation support, (5) mechanical ventilation initiated within 48 h prior to enrollment, and (6) the patient or his/her legal representative agreed to participate in the study and signed the informed consent form.

Exclusion criteria were as follows: (1) patients with a history of chronic respiratory diseases (such as chronic obstructive pulmonary disease and bronchial asthma), (2) patients with severe cardiovascular diseases (such as acute myocardial infarction and severe heart failure), (3) patients with acute diseases that affect respiratory function, such as pulmonary embolism or pneumothorax, (4) pregnant or lactating women, (5) patients who have recently (within 3 months) participated in other clinical trials, (6) patients with contraindications to EPM (such as esophageal varices and esophageal tumors), and (7) patients with an expected survival period less than 28 days.

The following baseline characteristics were collected at enrollment: demographic data, body mass index, comorbidities, etiology of SAP, acute physiology and chronic health evaluation II (APACHE II) score, sequential organ failure assessment (SOFA) score, modified Marshall score for SAP severity, vital signs, laboratory tests, vasopressor requirements, and initial ventilator settings.

Outcome assessors remained blinded to group allocation throughout the study. Given the nature of the intervention, blinding of treating physicians was not feasible. However, standardized protocols were followed for both groups to minimize potential bias.

### 2.2 Methods

#### 2.2.1 Common Management Protocol

All enrolled patients were mechanically ventilated using Hamilton G5 ventilators (Hamilton Medical AG, Switzerland). During the initial phase, all patients received volume‐controlled ventilation (VCV) with a tidal volume of 6 mL/kg predicted body weight (PBW). The respiratory rate was adjusted to maintain arterial carbon dioxide partial pressure (PaCO2) between 35 and 50 mmHg. The inspiratory to expiratory ratio

was set at 1:1.5 to 1:2. Initial positive end‐expiratory pressure (PEEP) and FiO2 were adjusted according to the ARDSNet low PEEP/high FiO2 table.

Pes was monitored in both groups using specialized esophageal balloon catheters (SmartCath, CareFusion, USA). These multipurpose catheters were also used for enteral feeding, which provided clinical justification for placement in both groups. The research team, consisting of six trained investigators, was available 24/7 in rotating shifts to perform measurements. In the conventional group, Pes data were collected for research purposes only and were concealed from the clinical team using a separate monitoring device that was password‐protected and only accessible to research personnel. Measurements were recorded at predetermined time points (baseline, 12, 24, 48, and 72 h) and stored for later analysis.

Neuromuscular blocking agents (cisatracurium, 0.15–0.2 mg/kg/h continuous infusion) were administered according to standardized protocol in both groups when indicated for patient–ventilator dyssynchrony or severe hypoxemia (PaO2/FiO2 < 150 mmHg) for the first 48 h of mechanical ventilation, after which daily interruption was attempted.

#### 2.2.2 Conventional Group Protocol

In the conventional group, patients received standard lung‐protective ventilation strategy. The ventilator parameters were adjusted to maintain plateau pressure (Pplat) ≤ 30 cmH2O and driving pressure (ΔP) ≤ 15 cmH2O. When Pplat exceeded 30 cmH2O, tidal volume was gradually reduced to 4 mL/kg PBW. PEEP and FiO2 adjustments followed the ARDSNet high PEEP/FiO2 table with the goal of maintaining oxygenation index (PaO2/FiO2 ratio) > 150 mmHg and arterial oxygen saturation (SaO2) > 88%. If respiratory acidosis (pH < 7.15) developed with tidal volumes of 4–6 mL/kg PBW, Pplat targets were increased to 32–35 cmH2O as a rescue strategy.

The ΔP target of ≤ 15 cmH2O was pursued primarily through tidal volume adjustments rather than PEEP modifications, as PEEP changes were dictated by oxygenation requirements according to the ARDSNet protocol. When ΔP exceeded 15 cmH2O, tidal volume was reduced in 0.5 mL/kg PBW increments to a minimum of 4 mL/kg PBW, with respiratory rate increases to maintain minute ventilation as tolerated. The adjustments were performed every 4 h or more frequently if clinically indicated.

#### 2.2.3 EPM‐Guided Group Protocol

In the EPM‐guided group, ventilation parameters were individualized based on EPM. The esophageal balloon catheter was inserted through the nose to a depth of 40–45 cm and then withdrawn to 30–35 cm for optimal positioning in the mid‐esophagus, with position confirmation by chest X‐ray and the closed airway test. The correct positioning was verified by observing cardiac oscillations on the Pes tracing and by performing an occlusion test (the ratio of changes in Pes to airway pressure during occluded respiratory efforts should be 0.8–1.2). The balloon was inflated with 0.5–2.0 mL of air, with the volume titrated to obtain the highest amplitude of cardiac oscillations without damping effect.

The Pes measurements were obtained by connecting the esophageal balloon catheter to the ventilator pressure sensor. Measurements were recorded every 2 h during the first 24 h, then every 4 h thereafter. The PL was calculated using the formula: PL = Paw—Pes, where Paw represents the airway pressure. End‐inspiratory PL was calculated as Pplat minus end‐inspiratory Pes, whereas end‐expiratory PL was calculated as PEEP minus end‐expiratory Pes.

The ventilation strategy in the EPM‐guided group followed these principles:

For tidal volume adjustment, the initial setting was 6 mL/kg PBW, with subsequent modifications based on PL measurements. The target was to maintain end‐inspiratory PL below 20 cmH2O. When this threshold was exceeded, tidal volume was progressively decreased to a minimum of 4 mL/kg PBW.

PEEP optimization was achieved through a decremental PEEP test. Starting from 15 cmH2O, PEEP was reduced by 2 cmH2O increments every 2 min until reaching 5 cmH2O. At each PEEP level, end‐expiratory PL, oxygenation index, and respiratory system compliance (Crs) were documented. The optimal PEEP was identified as the level maintaining end‐expiratory PL between 0 and 2 cmH2O with the highest Crs.

Lung recruitment maneuvers were performed when severe hypoxemia (PaO2/FiO2 ratio < 150 mmHg) or atelectasis (indicated by significant Crs decrease) occurred. The recruitment process involved a stepwise increase in PEEP to 30–40 cmH2O for 30–40 s, with continuous hemodynamic monitoring. Postrecruitment PEEP was set to the previously determined optimal level.

#### 2.2.4 Ventilator Parameter Control and Monitoring

In both groups, arterial blood gas analysis and respiratory mechanics evaluations were conducted at baseline, 12, 24, 48, and 72 h after initiation of mechanical ventilation. The ΔP was maintained ≤ 15 cmH2O, and in the EPM‐guided group, the ΔPL was kept ≤ 12 cmH2O. When these targets were exceeded, tidal volume reduction was prioritized, with respiratory rate adjustments as needed to maintain minute ventilation. Tidal volume (mL/kg PBW) was recorded at each time point for both groups.

#### 2.2.5 Weaning Protocol

Weaning readiness was assessed daily in both groups. Patients were considered for weaning when they met the following criteria for 24 consecutive hours: PaO2/FiO2 ratio > 200 mmHg, PEEP ≤ 8 cmH2O, FiO2 ≤ 0.5, pH > 7.30, and respiratory rate ≤ 35 breaths/min. A spontaneous breathing trial was conducted using pressure support ventilation mode for 30–120 min. Successful completion of the trial led to endotracheal tube removal.

#### 2.2.6 Posture Management

All patients were maintained in a 45° semirecumbent position. Prone positioning was implemented for patients with the PaO2/FiO2 ratio < 150 mmHg, with each session lasting at least 16 h. After each position change from supine to prone or vice versa, the esophageal balloon position was reconfirmed using the closed airway occlusion test, and the balloon volume was readjusted if necessary. Balloon position was also reconfirmed by chest X‐ray after the first proning session. The decision to initiate, continue, or terminate prone positioning was based on the patient's oxygenation response and clinical condition.

#### 2.2.7 Fluid Management Strategy

A conservative fluid management strategy was implemented in both groups according to a standardized protocol. The target was to maintain a neutral or slightly negative fluid balance after the initial resuscitation phase, while ensuring adequate perfusion (mean arterial pressure ≥ 65 mmHg and urine output ≥ 0.5 mL/kg/h). Daily fluid input, output, and cumulative fluid balance were recorded. Crystalloid solutions were preferentially used, and colloids were administered only when specifically indicated. Albumin was administered when serum albumin levels were < 25 g/L.

#### 2.2.8 Rescue Therapies

In cases of refractory hypoxemia (PaO2/FiO2 < 80 mmHg for > 6 h despite optimized ventilation, prone positioning, and neuromuscular blockade), rescue therapies were considered. Extracorporeal CO2 removal (ECCOR) was available as first‐line rescue therapy, followed by venovenous extracorporeal membrane oxygenation (VV‐ECMO) for eligible patients. No crossover between treatment groups was permitted, even in deteriorating patients, to maintain the integrity of the randomized design. The decision to initiate rescue therapies was made by a senior intensivist not involved in the study based on preestablished criteria.

### 2.3 Observation Indicators

The baseline values before treatment and the changes of various indicators at 12, 24, 48, and 72 h after treatment were recorded. The following indicators were mainly observed: (1) EPM indicators: including Pes (measured at end‐expiration), PL (calculated at both end‐inspiration and end‐expiration), and ΔPL (defined as the difference between end‐inspiratory and end‐expiratory PL). (2) Respiratory mechanics indicators: including PEEP, peak inspiratory pressure (PIP), Pplat, ΔP, tidal volume (VT, expressed in mL/kg PBW), and static compliance (Cst). (3) Lung compliance (CL, calculated as VT divided by ΔPL). (4) Oxygenation indicators: including oxygenation index (PaO2/FiO2 ratio) and arterial oxygen partial pressure (PaO2).

Clinical outcomes of the two groups of patients were evaluated, including mechanical ventilation duration, ICU length of stay, ventilator‐associated pneumonia (VAP) incidence, and 28‐day mortality rate. Adverse events during treatment were fully recorded in the two groups of patients.

### 2.4 Statistical Methods

SPSS 26.0 statistical software was used for data analysis. The Shapiro–Wilk test was used to determine whether the measurement data conformed to the normal distribution. The measurement data that conformed to the normal distribution were expressed as mean ± standard deviation (Mean ± SD), and the independent sample *t* test was used for intergroup comparison; the measurement data that did not conform to the normal distribution were expressed as median (interquartile range) [M(IQR)], and the Mann–Whitney *U* test was used for intergroup comparison. The count data were expressed as counts (percentage) [*n* (%)], and the chi‐squared test was used for intergroup comparison. Repeated measures ANOVA was used for repeated measurement data. For the multivariate logistic regression analysis, variables with *p* < 0.10 in univariate analyses were included in the model. Backward stepwise selection with the likelihood ratio test was used for variable selection in the final model. All statistical tests were two‐tailed, and *p* < 0.05 was considered statistically significant. This study followed the CONSORT guidelines for reporting randomized controlled trials.

## 3 Results

### 3.1 Comparison of Baseline Data

A total of 124 patients with SAP‐related ARDS completed the study, including 70 males and 54 females with an average age of (55.02 ± 12.13) years. As shown in Table 1, there were no significant differences in baseline characteristics, such as age, sex, body mass index (BMI), comorbidities, APACHE II score, and SOFA score, between the two groups (all *p* > 0.05).

**TABLE 1:** Comparison of baseline data of patients between the two groups (x̄ ± s).

Characteristics	EPM‐guided group (*n* = 62)	Conventional group (*n* = 62)	*t*/χ2 value	*p* value
Age (years)	54.73 ± 12.32	55.31 ± 11.94	0.276	0.783
Sex [*n* (%)]			0.142	0.706
Male	36 (58.06)	34 (54.84)		
Female	26 (41.94)	28 (45.16)		
BMI (kg/m2)	24.82 ± 3.43	24.56 ± 3.21	0.432	0.667
Comorbidities [*n* (%)]				
Hypertension	21 (33.87)	19 (30.65)	0.156	0.693
Diabetes	15 (24.19)	17 (27.42)	0.173	0.677
Coronary heart disease	12 (19.35)	11 (17.74)	0.058	0.810
APACHE II score	18.43 ± 4.62	18.76 ± 4.83	0.392	0.696
SOFA score	8.92 ± 2.34	8.76 ± 2.41	0.374	0.709
Modified Marshall score	5.7 ± 1.2	5.8 ± 1.3	0.456	0.649
Etiology of SAP [*n* (%)]			0.568	0.904
Gallstone‐related	27 (43.5)	25 (40.3)		
Alcohol‐induced	16 (25.8)	18 (29.0)		
Hypertriglyceridemia	12 (19.4)	10 (16.1)		
Other causes	7 (11.3)	9 (14.5)		
Vasopressor use [*n* (%)]	29 (46.8)	30 (48.4)	0.033	0.856
Neuromuscular blockade use [*n* (%)]	36 (58.1)	39 (62.9)	0.296	0.586

Abbreviations: APACHE II score, acute physiology and chronic health evaluation II score; BMI, body mass index; EPM, esophageal pressure monitoring; SOFA score, sequential organ failure assessment score.

### 3.2 Changes in Esophageal Pressure Monitoring Indicators

Before treatment, there were no significant differences in PL, ΔPL, and Pes between the two groups (all *p* > 0.05). After 24 h of treatment, end‐inspiratory PL in the EPM‐guided group began to be significantly lower than that in the conventional group [(19.42 ± 2.83) versus. (22.52 ± 3.24) cmH2O, *p* = 0.012]. ΔPL (defined as the difference between end‐inspiratory and end‐expiratory PL) in the EPM‐guided group also showed a continuous downward trend, reaching the lowest value at 72 h [(12.36 ± 1.83) versus. (16.52 ± 2.37) cmH2O, *p* = 0.007]. The change in end‐expiratory Pes was relatively mild, but the EPM‐guided group was still significantly lower than the conventional group (Table 2). All indicators in the two groups were significantly improved compared to baseline (*p* < 0.05), and the improvement in the EPM‐guided group was more significant.

**TABLE 2:** Comparison of esophageal pressure monitoring indicators between the two groups at different time points (Mean ± SD, cmH2O).

Indicators	Time point	EPM‐guided group (*n* = 62)	Conventional group (*n* = 62)	*t* value	*p* value
End‐inspiratory PL	Baseline	22.86 ± 3.42	22.73 ± 3.38	0.213	0.832
	24 h	19.42 ± 2.83^ab^	22.52 ± 3.24	5.624	0.012
	48 h	17.63 ± 2.62^ab^	22.38 ± 3.18	6.326	0.008
	72 h	16.82 ± 2.46^ab^	22.41 ± 3.23	7.124	0.006
End‐expiratory PL	Baseline	6.13 ± 1.82	6.15 ± 1.78	0.062	0.951
	24 h	5.10 ± 1.63^ab^	6.09 ± 1.82	3.021	0.037
	48 h	4.39 ± 1.35^ab^	5.90 ± 1.73	5.236	0.014
	72 h	4.46 ± 1.38^ab^	5.89 ± 1.81	4.852	0.020
ΔPL	Baseline	16.73 ± 2.52	16.58 ± 2.43	0.342	0.733
	24 h	14.32 ± 2.13^ab^	16.43 ± 2.38	5.123	0.015
	48 h	13.24 ± 1.92^ab^	16.48 ± 2.42	6.234	0.009
	72 h	12.36 ± 1.83^ab^	16.52 ± 2.37	6.843	0.007
End‐expiratory pes	Baseline	12.83 ± 2.42	12.76 ± 2.38	0.162	0.872
	24 h	11.42 ± 2.13^ab^	12.62 ± 2.32	2.843	0.042
	48 h	10.86 ± 1.92^ab^	12.58 ± 2.28	3.126	0.038
	72 h	10.43 ± 1.83^ab^	12.52 ± 2.24	3.842	0.032

*Note:* Compared to baseline, a *p* < 0.05; compared to the conventional group, b *p* < 0.05.

Abbreviations: EPM, esophageal pressure monitoring; Pes, esophageal pressure; PL, transpulmonary pressure; ΔPL, transpulmonary driving pressure.

### 3.3 Changes in Respiratory Mechanics Parameters

As the treatment progressed, the respiratory mechanics parameters of the patients in the EPM‐guided group were significantly improved compared with the baseline values. As shown in Table 3, the Pplat in the EPM‐guided group decreased from (28.86 ± 3.42) cmH2O at baseline to (22.82 ± 2.46) cmH2O at 72 h (*p* < 0.05), which was significantly lower than the level of the conventional group during the same period. The change trend of ΔP was similar, the EPM‐guided group reached (11.43 ± 1.83) cmH2O at 72 h, whereas the conventional group remained at (14.52 ± 2.24) cmH2O (*p* = 0.008). It is worth noting that the intergroup difference in PEEP was primarily observed in the early stage of treatment (24 and 48 h), after which the values became more comparable. The Cst of the EPM‐guided group continued to improve, reaching (37.82 ± 4.46) mL/cmH2O at 72 h, which was significantly higher than that of the conventional group (29.41 ± 5.23) mL/cmH2O (*p* = 0.009).

**TABLE 3:** Comparison of respiratory mechanics parameters between the two groups at different time points (x̄ ± s).

Parameter	Time point	EPM‐guided group (*n* = 62)	Conventional group (*n* = 62)	*t* value	*p* value
Pplat (cmH_2_O)	Baseline	28.86 ± 3.42	28.73 ± 3.38	0.213	0.832
	24 h	25.42 ± 2.83^ab^	28.52 ± 3.24	5.624	0.013
	48 h	23.63 ± 2.62^ab^	28.38 ± 3.18	6.326	0.008
	72 h	22.82 ± 2.46^ab^	28.41 ± 3.23	7.124	0.006
PEEP (cmH_2_O)	Baseline	8.73 ± 2.52	8.58 ± 2.43	0.342	0.733
	24 h	12.32 ± 2.13^ab^	10.43 ± 2.38	4.123	0.026
	48 h	11.24 ± 1.92^ab^	10.48 ± 2.42	2.234	0.048
	72 h	10.36 ± 1.83^a^	10.52 ± 2.37	0.843	0.401
ΔP (cmH_2_O)	Baseline	14.83 ± 2.42	14.76 ± 2.38	0.162	0.872
	24 h	12.42 ± 2.13^ab^	14.62 ± 2.32	5.843	0.016
	48 h	11.86 ± 1.92^ab^	14.58 ± 2.28	6.126	0.011
	72 h	11.43 ± 1.83^ab^	14.52 ± 2.24	6.842	0.008
VT (mL/kg PBW)	Baseline	6.01 ± 0.12	6.02 ± 0.14	0.432	0.667
	24 h	5.82 ± 0.24^ab^	6.00 ± 0.18	4.736	0.021
	48 h	5.64 ± 0.32^ab^	5.98 ± 0.32	5.128	0.013
	72 h	5.43 ± 0.38^ab^	5.92 ± 0.42	6.524	0.006
Cst (mL/cmH_2_O)	Baseline	29.83 ± 5.42	29.76 ± 5.38	0.072	0.943
	24 h	33.42 ± 4.83^ab^	29.52 ± 5.24	4.624	0.024
	48 h	35.63 ± 4.62^ab^	29.38 ± 5.18	5.326	0.015
	72 h	37.82 ± 4.46^ab^	29.41 ± 5.23	6.124	0.009
CL (mL/cmH_2_O)	Baseline	35.86 ± 4.82	36.12 ± 4.97	0.298	0.767
	24 h	40.64 ± 5.12^ab^	36.52 ± 4.87	4.213	0.027
	48 h	42.60 ± 5.18^ab^	36.28 ± 4.93	5.842	0.013
	72 h	44.32 ± 5.26^ab^	35.81 ± 4.98	6.738	0.007

*Note:* Compared to baseline, a *p* < 0.05; compared to the conventional group, b *p* < 0.05.

Abbreviations: CL, lung compliance; Cst, static compliance of respiratory system; EPM, esophageal pressure monitoring; ΔP, driving pressure; PBW, predicted body weight; PEEP, positive end‐expiratory pressure; Pplat, plateau pressure; VT, tidal volume.

In terms of tidal volume, the EPM‐guided group showed a progressive decrease from baseline (6.01 ± 0.12) mL/kg PBW to (5.43 ± 0.38) mL/kg PBW at 72 h, whereas the conventional group maintained higher tidal volumes throughout the study period, ranging from (6.02 ± 0.14) to (5.92 ± 0.42) mL/kg PBW at 72 h (*p* = 0.006). This difference in tidal volume management likely contributed to the observed differences in ΔP between the groups.

CL, calculated as VT divided by ΔPL, was significantly higher in the EPM‐guided group compared to the conventional group at 72 h [(44.32 ± 5.26) versus. (35.81 ± 4.98) mL/cmH2O, *p* = 0.007], indicating better preserved lung mechanics with the Pes‐guided strategy.

The higher driving pressures observed in the conventional group (frequently exceeding the safety threshold of 15 cmH2O) were primarily due to the combination of relatively higher tidal volumes and lower compliance in this group as the protocol permitted tidal volumes up to 6 mL/kg PBW as long as Pplat remained ≤ 30 cmH2O.

### 3.4 Improvement of Oxygenation Function

The oxygenation function of the two groups of patients improved to varying degrees after treatment (Table 4). The primary outcome measure, PaO2/FiO2 ratio, showed a significant improvement in both groups from baseline, with a between‐group difference of 73.41 ± 12.42 mmHg at 72h in favor of the EPM‐guided group. The PaO2/FiO2 ratio of the EPM‐guided group showed a significant advantage after 24 h [(186.42 ± 28.83) versus. (165.52 ± 27.24) mmHg, *p* = 0.028]. At 72 h, the EPM‐guided group reached (268.82 ± 32.46) mmHg, which was significantly higher than the (195.41 ± 28.23) mmHg of the conventional group (*p* = 0.008). The improvement trend of arterial oxygen partial pressure and blood oxygen saturation was similar to that of the oxygenation index and the EPM‐guided group showed better improvement (all *p* < 0.05).

**TABLE 4:** Comparison of oxygenation indicators between two groups at different time points (x̄ ± s).

Indicator	Time point	EPM‐guided group (*n* = 62)	Conventional group (*n* = 62)	*t* value	*p* value
PaO2/FiO2 ratio (mmHg)	Baseline	142.86 ± 32.42	143.73 ± 33.38	0.143	0.887
	24 h	186.42 ± 28.83^ab^	165.52 ± 27.24	4.124	0.028
	48 h	223.63 ± 30.62^ab^	182.38 ± 28.18	5.326	0.016
	72 h	268.82 ± 32.46^ab^	195.41 ± 28.23	6.224	0.008
PaCO2 (mmHg)	Baseline	46.8 ± 5.9	47.2 ± 6.1	0.374	0.709
	24 h	43.5 ± 4.8^a^	42.9 ± 5.2^a^	0.684	0.496
	48 h	42.1 ± 4.5^a^	41.2 ± 4.3^a^	1.162	0.248
	72 h	41.2 ± 4.3a	40.8 ± 4.1^a^	0.509	0.612
pH	Baseline	7.31 ± 0.06	7.30 ± 0.07	0.862	0.391
	24 h	7.34 ± 0.05^a^	7.33 ± 0.06^a^	1.026	0.307
	48 h	7.36 ± 0.04^a^	7.35 ± 0.05^a^	1.224	0.224
	72 h	7.38 ± 0.04^a^	7.37 ± 0.05^a^	0.308	0.758

*Note:* Compared to baseline, a *p* < 0.05; compared to the conventional group, b *p* < 0.05.

Abbreviations: EPM, esophageal pressure monitoring; PaO2/FiO2, oxygenation index; PaO2, arterial oxygen partial pressure; PaCO2, arterial carbon dioxide partial pressure; SaO2, arterial oxygen saturation.

### 3.5 Comparison of Fluid Management and Balance

The cumulative fluid balance at 72 h was slightly negative in the EPM‐guided group [(−352 ± 628) mL] compared to a positive balance in the conventional group [(+483 ± 712) mL] (*p* = 0.042). Daily fluid intake was similar between groups, but the EPM‐guided group had higher urinary output from 48 h onward, likely reflecting improved hemodynamic stability and renal perfusion. There were no significant differences in the use of vasopressors or inotropes between the two groups (Table 5).

**TABLE 5:** Fluid balance comparison between the two groups (x̄ ± s).

Parameter	Time point	EPM‐guided group (*n* = 62)	Conventional group (*n* = 62)	*t* value	*p* value
Daily fluid intake (mL)	Day 1	2842 ± 483	2924 ± 512	0.932	0.353
	Day 2	2638 ± 432	2752 ± 467	1.382	0.169
	Day 3	2473 ± 398	2512 ± 423	0.528	0.598
Daily fluid output (mL)	Day 1	2463 ± 512	2382 ± 483	0.914	0.362
	Day 2	2748 ± 463^b^	2483 ± 428	3.268	0.038
	Day 3	2954 ± 487^b^	2520 ± 459	5.153	0.016
Daily fluid balance (mL)	Day 1	379 ± 216	542 ± 283	3.642	0.035
	Day 2	−110 ± 187^b^	269 ± 243	4.837	0.022
	Day 3	−481 ± 232^b^	−8±198	5.926	0.011
Cumulative fluid balance (mL)	24 h	379 ± 216^b^	542 ± 283	3.642	0.035
	48 h	269 ± 382^b^	811 ± 497	6.438	0.009
	72 h	−352 ± 628^b^	483 ± 712	7.132	0.005

*Note:* Compared to the conventional group, b *p* < 0.05.

Abbreviation: EPM, esophageal pressure monitoring.

### 3.6 Correlation Analysis

**FIGURE 1:**
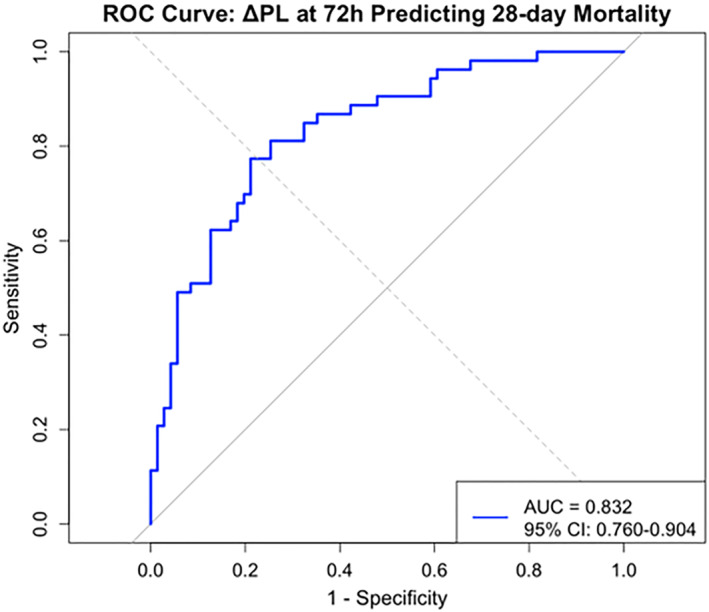
Correlation heat map.

Correlation analysis between parameters showed (Figure 1) that ΔPL at 72 h was significantly negatively correlated with the PaO2/FiO2 ratio (*r* = −0.71 and *p* < 0.001) and static compliance (*r* = −0.69 and *p* < 0.001). PL was moderately positively correlated with ΔPL (*r* = 0.72 and *p* < 0.001). PaO2/FiO2 ratio was positively correlated with static compliance (*r* = 0.65 and *p* < 0.001). Additionally, CL showed a stronger correlation with the PaO2/FiO2 ratio (*r* = 0.78 and *p* < 0.001) than respiratory system compliance, highlighting the physiological relevance of PL‐based measurements in this patient population.

### 3.7 Predictors of 28‐day Mortality Rate

Multivariate logistic regression analysis showed that after adjusting for confounding factors, such as age and APACHE II score, ΔPL at 72 h (OR 1.56, 95% CI 1.25–2.01, and *p* < 0.001) was an independent predictor of a 28‐day mortality rate, whereas other factors, such as age (OR 1.02, 95% CI 0.98–1.07, and *p* = 0.282), APACHE II score (OR 1.01, 95% CI 0.92–1.11, and *p* = 0.841), and EPM treatment strategy (OR 0.63, 95% CI 0.08–4.73, and *p* = 0.653), were not significantly correlated with prognosis (Table 6).

**TABLE 6:** Multivariate logistic regression analysis of a 28‐day mortality rate.

Variable	*β* coefficient	Standard error	Wald *χ*2 value	OR value	95% CI	*p* value
Constant	−5.27	1.62	10.58	—	—	0.001
ΔPL	0.445	0.127	12.31	1.56	1.25–2.01	< 0.001
PL	0.139	0.089	2.43	1.15	0.97–1.38	0.122
PaO2/FiO2 ratio	0.009	0.008	1.44	1.01	0.99–1.03	0.231
Static compliance	−0.0002	0.051	0.00	1.00	0.91–1.10	0.997
Age	0.023	0.022	1.16	1.02	0.98–1.07	0.282
APACHE II score	0.010	0.049	0.04	1.01	0.92–1.11	0.841
EPM treatment strategies	−0.459	1.024	0.20	0.63	0.08–4.73	0.653

Abbreviations: EPM, esophageal pressure monitoring; PaO2/FiO2, oxygenation index; ΔPL, transpulmonary driving pressure; PL, transpulmonary pressure.

It is important to note that although PL was used both as an intervention parameter and an outcome measure in this study, the multivariate analysis identifies ΔPL as an independent predictor of mortality regardless of the treatment group, suggesting its intrinsic physiological significance beyond its role in the intervention.

**FIGURE 2:**
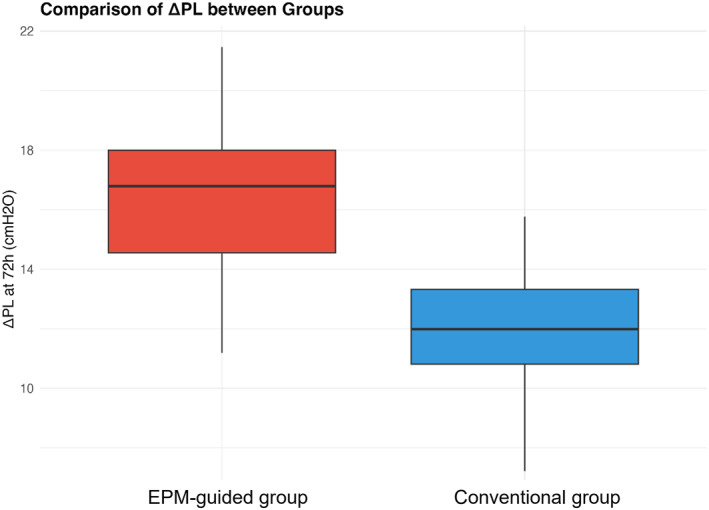
ROC curve: ΔPL predicts a 28‐day mortality rate.

**FIGURE 3:**
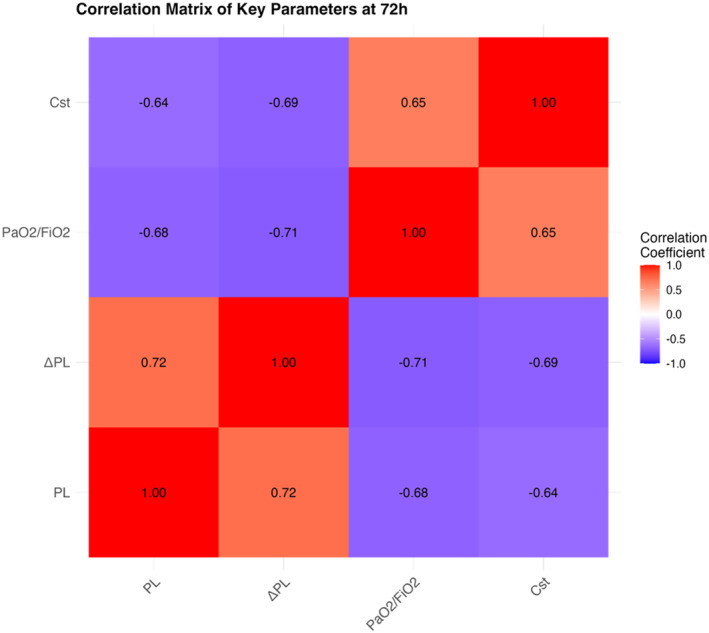
Boxplot of ΔPL comparison between the two groups.

ROC curve analysis showed that ΔPL at 72 h had a good diagnostic value for predicting a 28‐day mortality rate (AUC = 0.832 and 95% CI 0.760–0.904) (Figure 2). When ΔPL increased, the patient's risk of a 28‐day mortality rate increased significantly. The ΔPL in the EPM‐guided group was significantly lower than that in the conventional group (Figure 3), suggesting that this strategy may improve prognosis by reducing ΔPL.

### 3.8 Clinical Outcomes

The EPM‐guided group demonstrated superior clinical outcomes compared to the conventional group (Table 7). The EPM‐guided group had shorter mechanical ventilation duration [(12.32 ± 3.24) versus. (16.83 ± 4.52) d, *p* = 0.013] and ICU length of stay [(18.53 ± 4.62) versus. (23.72 ± 5.83) d, *p* = 0.018] compared to the conventional group, along with a lower VAP incidence (14.52% vs. 25.81%, *p* = 0.038) and a 28‐day mortality rate (19.35% vs. 32.26%, *p* = 0.042). The EPM‐guided group also showed higher weaning success rates (85.48% vs. 74.19%, *p* = 0.032), lower first weaning failure rates (19.35% vs. 29.03%, *p* = 0.042), and reduced tracheotomy rates (24.19% vs. 33.87%, *p* = 0.048). No rescue therapies (ECCOR or VV‐ECMO) were required in either group during the study period.

**TABLE 7:** Comparison of clinical prognostic indicators between the two groups.

Indicator	EPM‐guided group (*n* = 62)	Conventional group (*n* = 62)	*t* Value/χ2 value	*p* value
Mechanical ventilation duration (d x̄ ± s)	12.32 ± 3.24	16.83 ± 4.52	*t* = 5.824	0.013
ICU length of stay (d x̄ ± s)	18.53 ± 4.62	23.72 ± 5.83	*t* = 5.326	0.018
VAP incidence [*n* (%)]	9 (14.52)	16 (25.81)	*χ*2 = 4.324	0.038
28‐day mortality rate [*n* (%)]	12 (19.35)	20 (32.26)	*χ*2 = 4.128	0.042
Complications [*n* (%)]				
Stress ulcer	8 (12.90)	10 (16.13)	*χ*2 = 0.262	0.609
New‐onset arrhythmia	6 (9.68)	8 (12.90)	*χ*2 = 0.324	0.569
Acute kidney injury	7 (11.29)	9 (14.52)	*χ*2 = 0.282	0.595
Thrombosis	4 (6.45)	5 (8.06)	*χ*2 = 0.120	0.729
Weaning success rate [*n* (%)]	53 (85.48)	46 (74.19)	*χ*2 = 4.623	0.032
First weaning failure rate [*n* (%)]	12 (19.35)	18 (29.03)	*χ*2 = 4.126	0.042
Tracheotomy rate [*n* (%)]	15 (24.19)	21 (33.87)	*χ*2 = 3.924	0.048
Rescue therapies [*n* (%)]				
ECCOR	0 (0)	0 (0)	—	—
VV‐ECMO	0 (0)	0 (0)	—	—

Abbreviations: ECCOR, extracorporeal CO2 removal; EPM, esophageal pressure monitoring; VAP, ventilator‐associated pneumonia; VV‐ECMO, venovenous extracorporeal membrane oxygenation.

## 4 Discussion

This randomized controlled trial evaluated the application value of individualized lung protective ventilation strategy based on EPM in patients with SAP‐related ARDS. The results showed that compared with the traditional lung protective ventilation strategy, the individualized ventilation strategy guided by EPM can more accurately assess the actual lung inflation pressure, optimize the ventilator parameter settings, and improve the oxygenation function and clinical outcomes of patients.

### 4.1 Key Findings and Comparison With Previous Studies

The study found that patients in the EPM‐guided group showed significantly reduced PL and ΔPL early in the treatment. This finding is consistent with the results of Talmor et al., who found in a randomized controlled trial that a strategy of adjusting PEEP based on EPM can maintain PL within a safe range [12]. Studies have shown that for every 1 mmHg increase in intra‐abdominal pressure, the intrathoracic pressure will increase by 0.5–0.8 mmHg [13]. Therefore, for patients with SAP‐ARDS, the traditional ventilation strategy based solely on airway pressure may underestimate the actual alveolar stress.

However, our results differ from those reported by Beitler et al. in their 2019 JAMA study, which found no significant differences in mortality or ventilator‐free days between an Pes‐guided strategy and a high‐PEEP empirical strategy in a general ARDS population [14]. Several factors may explain these discrepancies. First, our study specifically focused on SAP‐related ARDS, a condition characterized by significant intra‐abdominal hypertension that may particularly benefit from PL‐guided ventilation. Second, Beitler et al. used a high‐PEEP empirical strategy in their control group, whereas we used the ARDSNet high PEEP/FiO2 table, which may not be as effective in addressing the specific pathophysiology of SAP‐related ARDS. Third, our sample size calculation was based on the PaO2/FiO2 ratio rather than mortality, reflecting our focus on the physiological effects of the intervention. Despite this, we observed a significant mortality benefit, which may be attributed to the homogeneous nature of our study population with a shared underlying pathophysiology.

A systematic review and meta‐analysis showed that mechanical ventilation strategies based on PL can significantly improve the oxygenation index and CL of patients with ARDS and reduce the duration of mechanical ventilation and the mortality rate of patients [6]. This meta‐analysis included seven randomized controlled trials with a total of 517 patients, showing a consistent benefit of PL‐guided ventilation, particularly in patients with extrapulmonary causes of ARDS, which aligns with our findings in SAP‐related ARDS.

Excessive PL can lead to alveolar overdistension and shear injury, triggering inflammatory responses and aggravating lung damage. Studies have confirmed that sustained high PL activates mechanosensitive ion channels, leading to increased calcium ion influx, triggering cell pyroptosis and the release of inflammatory factors [15]. This study achieved accurate assessment of actual alveolar pressure through EPM, avoiding the pressure assessment bias that may be caused by traditional methods.

### 4.2 Respiratory Mechanics and Ventilation Strategy

In terms of respiratory mechanics, patients in the EPM‐guided group showed significant improvement compared with the conventional group, especially in static compliance. This improvement may be due to multiple mechanisms: individualized PEEP setting helps prevent alveolar collapse and maintain appropriate functional residual capacity [16]; precisely controlled ΔP can avoid excessive alveolar expansion caused by excessive tidal volume [17]; and lung recruitment strategy based on EPM can more effectively open collapsed alveoli and improve the alveolar ventilation/perfusion ratio [18]. Studies have found that maintaining appropriate PL can not only improve the secretion of alveolar surfactant but also promote the repair of alveolar epithelial cells [19].

The progressive decrease in tidal volume observed in the EPM‐guided group (from 6.01 to 5.43 mL/kg PBW) without compromising ventilation parameters (PaCO2 and pH) suggests that PL‐guided ventilation may allow for more effective lung protection without the physiological consequences often associated with ultralow tidal volume strategies. This finding is particularly relevant as recent research has questioned whether the conventional 6 mL/kg PBW target is sufficiently protective in all patients with ARDS, with some benefiting from even lower volumes when guided by appropriate physiological measurements.

In this study, the improvement in oxygenation function of patients in the EPM‐guided group showed a significant time‐dependent characteristic and this improvement persisted in the later stage of treatment. This result is similar to the results of other studies [20]. Appropriate PL can promote the proliferation and differentiation of alveolar type II cells by activating mechanical force receptors, increase the synthesis and secretion of surfactant, and thus continuously improve alveolar function [21]. The continuous improvement of oxygenation function may be achieved through multiple mechanisms: appropriate PEEP levels promote alveolar recruitment and improve ventilation/perfusion matching; precisely controlled PL reduces lung damage and is conducive to lung function recovery; and individualized lung recruitment strategies can more effectively open collapsed alveoli and increase the effective gas exchange area [22, 23].

### 4.3 Clinical Outcomes

In terms of clinical outcomes, the EPM‐guided group had shorter mechanical ventilation duration, ICU length of stay, and lower rates of VAP and 28‐day mortality, which aligns with some previous studies [24]. These improvements in clinical outcomes are particularly notable given that our multivariate analysis did not identify EPM treatment strategy as an independent predictor of 28‐day mortality (OR 0.63, 95% CI 0.08–4.73, and *p* = 0.653). This apparent paradox can be explained by the fact that the beneficial effect of the EPM strategy is likely mediated through its physiological effects, particularly the reduction in ΔPL, rather than being an independent factor. The EPM strategy leads to improved lung mechanics and gas exchange, which in turn results in better clinical outcomes. This interpretation is supported by our finding that ΔPL at 72 h was an independent predictor of mortality (OR 1.56, 95% CI 1.25–2.01, and *p* < 0.001), serving as a mediator between the intervention and the clinical outcome.

The improved prognosis may be due to multiple aspects: precise pressure control reduces the occurrence of ventilator‐induced lung injury (VILI), good oxygenation function improves tissue perfusion and reduces the risk of organ dysfunction, and shorter mechanical ventilation duration also effectively reduces the occurrence of complications such as VAP. In addition, the EPM‐guided group showed a higher weaning success rate and a lower first weaning failure rate [25, 26]. Zhang et al. conducted a meta‐analysis of randomized controlled trials specifically examining mechanical ventilation guided by PL in patients with ARDS, which demonstrated that this approach was more effective than traditional lung protection ventilation strategies in improving oxygenation index and CL and reducing the number of days of mechanical ventilation [27].

### 4.4 Predictive Value of Transpulmonary Driving Pressure

This study determined through multivariate analysis that ΔPL at 72 h was an independent risk factor for predicting a 28‐day mortality rate (OR 1.56 and 95% CI 1.25–2.01). This suggests that ΔPL may be a key indicator reflecting the prognosis of patients with SAP‐ARDS. In addition, ROC curve analysis showed that ΔPL had a good predictive value (AUC = 0.832). It is important to clarify that ΔPL at 72 h in this analysis represents an outcome measurement reflecting the patient's physiological state after treatment rather than an intervention variable. Although the EPM treatment strategy itself was not identified as an independent predictor of mortality in our multivariate model, it appears to exert its beneficial effects through the reduction of ΔPL, which was significantly lower in the EPM‐guided group at 72 h. This finding aligns with the concept that mechanical power and energy transfer to the lung tissue are key determinants of VILI and subsequent outcomes, with ΔPL serving as a more physiologically relevant metric than airway ΔP in patients with altered chest wall mechanics.

Correlation analysis revealed that ΔPL was significantly negatively correlated with the PaO2/FiO2 ratio (*r* = −0.71), a finding that supports the direct link between PL and lung injury. Additionally, the negative correlation between ΔPL and static compliance (*r* = −0.69) suggests that excessive PL may cause lung injury by affecting the mechanical properties of lung tissue. Related in vitro experiments have confirmed that sustained high PL can lead to enhanced mechanical stress and inflammatory response of alveolar epithelial cells [28]. The positive correlation between the PaO2/FiO2 ratio and static compliance (*r* = 0.65) reveals the close relationship between alveolar recruitment status and oxygenation function, providing a theoretical basis for the formulation of individualized PEEP strategies.

### 4.5 Limitations

This study has several limitations that should be acknowledged. First, the dual role of PL as both an intervention parameter and outcome measure creates an inherent methodological bias, though it remains valuable for comparing actual lung stress between groups. Second, although our sample size was adequately powered for detecting differences in the PaO2/FiO2 ratio, it may have been underpowered for mortality differences and the observed mortality benefit should be interpreted with caution. Third, the single‐center design limits the external validity of our findings, particularly given the high incidence of SAP‐ARDS at our institution, which may not be representative of other settings. Fourth, biological indicators, such as pulmonary inflammatory markers, were not collected, preventing complete elucidation of underlying mechanisms. Fifth, the 28‐day follow‐up period limited assessment of long‐term outcomes. Additionally, the EPM technique itself has limitations, as Pes may not perfectly reflect pleural pressure in all lung regions, particularly in heterogeneously affected ARDS lungs. Despite these limitations, our findings suggest clear benefits of Pes‐guided ventilation in patents SAP‐related ARDS.

Our study also has several strengths. The focus on a specific ARDS subpopulation (SAP‐related) with a shared pathophysiology may have increased the likelihood of benefit from the intervention. The comprehensive assessment of both physiological parameters and clinical outcomes provides a complete picture of the effects of the intervention. The use of standardized protocols for both ventilation strategies and the blinded assessment of outcomes reduces the risk of bias.

In conclusion, the individualized lung protective ventilation strategy guided by EPM showed obvious advantages in patients with SAP‐related ARDS. This strategy can more accurately assess the actual lung inflation pressure, optimize the ventilation parameter settings, and improve the patient's respiratory mechanics and oxygenation function. These physiological improvements translate into meaningful clinical benefits, including reduced duration of mechanical ventilation, shorter ICU stay, and lower mortality. Although our findings differ from some previous studies in general ARDS populations, they suggest that targeting PL may be particularly beneficial in SAP‐related ARDS, where altered abdominal‐thoracic mechanics play a significant role in pathophysiology. In the future, a larger‐scale multicenter randomized controlled study is needed to further verify its clinical value.

## Author Contributions

**Yang Zhao:** conceptualization, data curation, formal analysis, investigation, methodology, writing – original draft, writing – review and editing. **Shengxuan Zhang**: conceptualization, data curation, formal analysis, investigation, methodology, writing – original draft, writing – review and editing. **Chengjiang Liu**: funding acquisition, project administration, resources, visualization, writing – review and editing. **Xiaoxia Wang:** funding acquisition, project administration, supervision, validation, visualization, writing – review and editing.

## Ethics Statement

This study was conducted in accordance with the ethical standards of the institutional and/or national research committee and with the 1964 Helsinki declaration and its later amendments or comparable ethical standards. The study protocol was approved by the Ethics Committee of Affiliated Anqing First People's Hospital of Anhui Medical University (AFky2023010322).

## Consent

The authors have nothing to report.

## Conflicts of Interest

The authors declare no conflicts of interest.

## Data Availability

All data generated or analyzed during this study are included in this published article.
